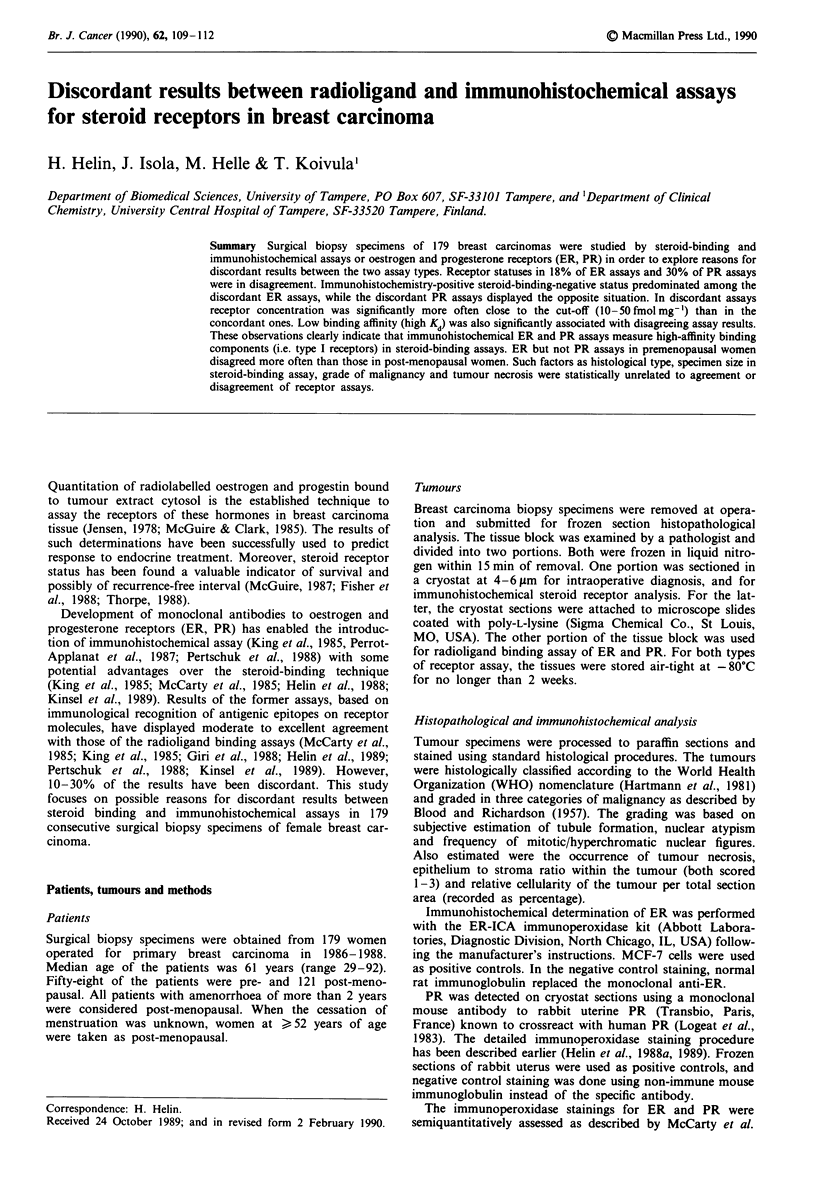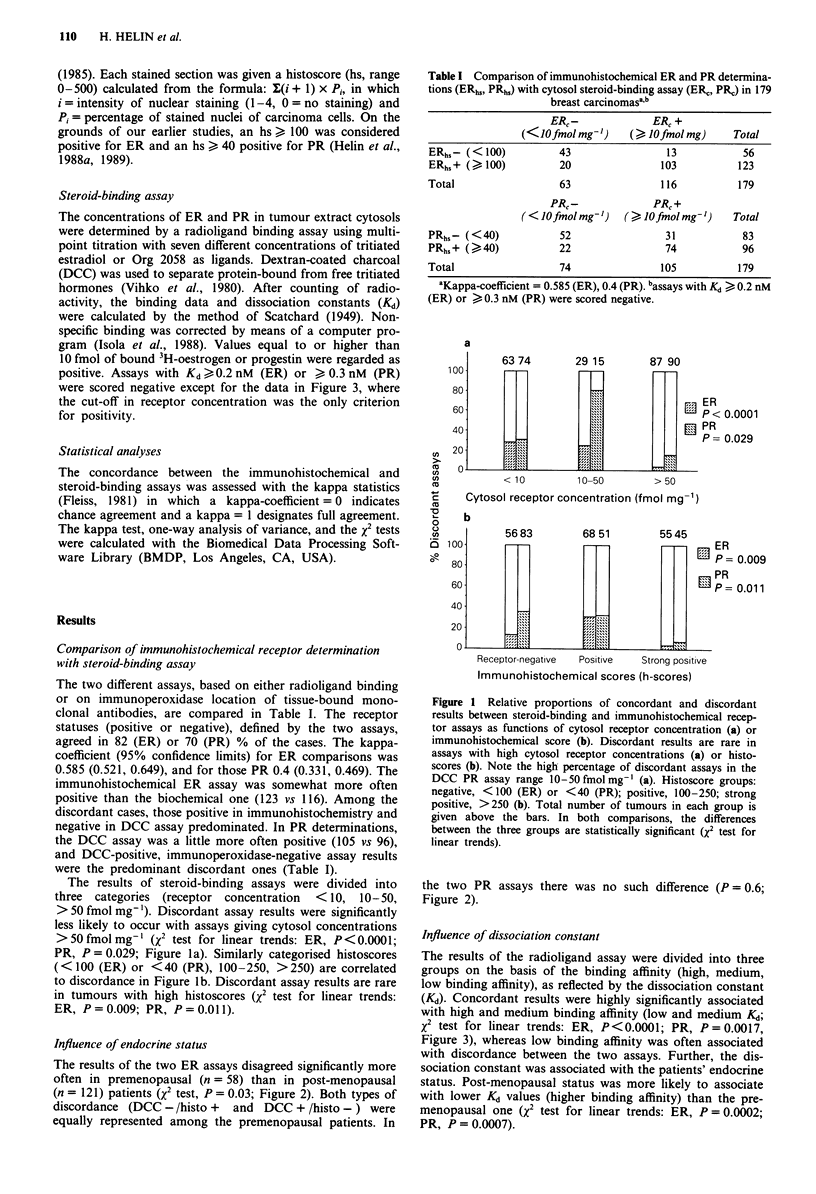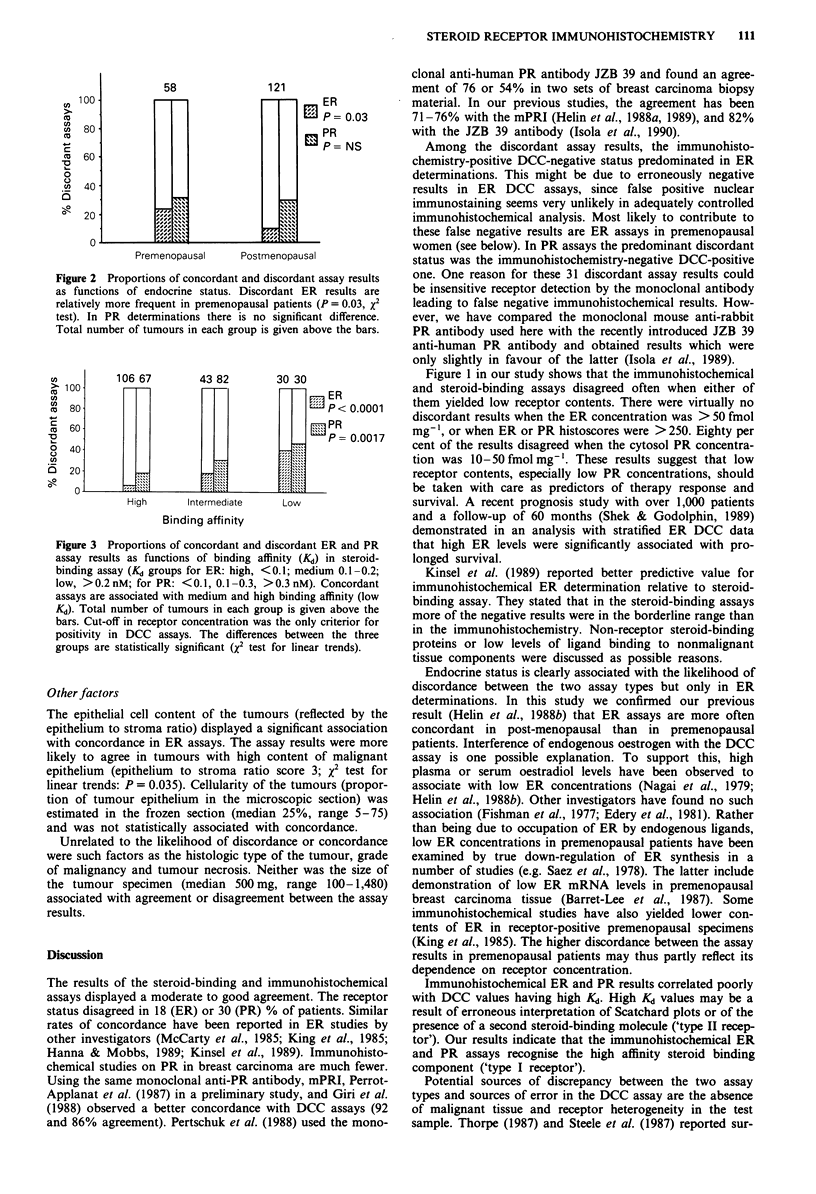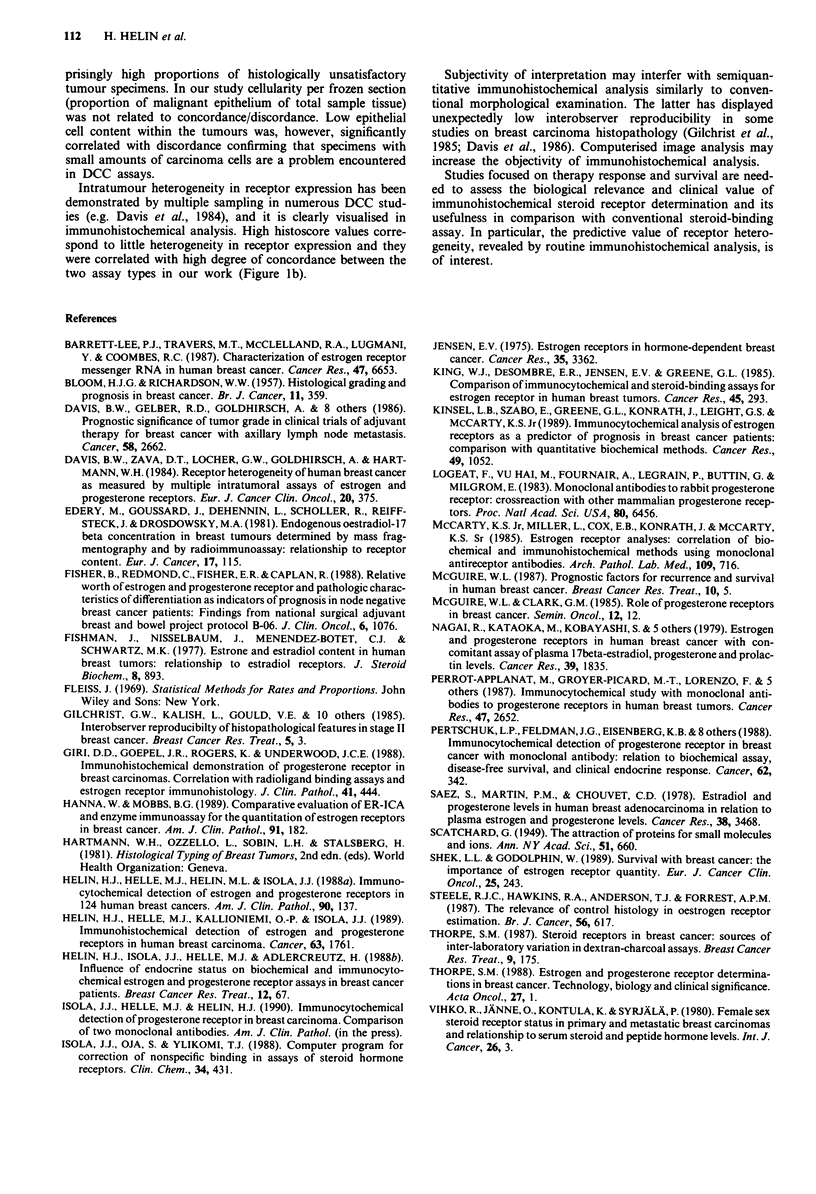# Discordant results between radioligand and immunohistochemical assays for steroid receptors in breast carcinoma.

**DOI:** 10.1038/bjc.1990.239

**Published:** 1990-07

**Authors:** H. Helin, J. Isola, M. Helle, T. Koivula

**Affiliations:** Department of Biomedical Sciences, University of Tampere, Finland.

## Abstract

Surgical biopsy specimens of 179 breast carcinoma were studied by steroid-binding and immunohistochemical assays or oestrogen and progesterone receptors (ER, PR) in order to explore reasons for discordant results between the two assay types. Receptor statuses in 18% of ER assays and 30% of PR assays were in disagreement. Immunohistochemistry-positive steroid-binding-negative status predominated among the discordant ER assays, while the discordant PR assays displayed the opposite situation. In discordant assays receptor concentration was significantly more often close to the cut-off (10-50 fmol mg-1) than in the concordant ones. Low binding affinity (high Kd) was also significantly associated with disagreeing assay results. These observations clearly indicate that immunohistochemical ER and PR assays measure high-affinity binding components (i.e. type I receptors) in steroid-binding assays. ER but not PR assays in premenopausal women disagreed more often than those in post-menopausal women. Such factors as histological type, specimen size in steroid-binding assay, grade of malignancy and tumour necrosis were statistically unrelated to agreement or disagreement of receptor assays.


*Br. J. Cancer* (1990), **62**, 109–112 © Macmillan Press Ltd., 1990

### Discordant results between radioligand and immunohistochemical assays for steroid receptors in breast carcinoma

H. Helin, J. Isola, M. Helle & T. Koivula[1]

*Department of Biomedical Sciences, University of Tampere, PO Box 607, SF-33101 Tampere, and [1]Department of Clinical Chemistry, University Central Hospital of Tampere, SF-33520 Tampere, Finland.*

**Summary** Surgical biopsy specimens of 179 breast carcinomas were studied by steroid-binding and immunohistochemical assays or oestrogen and progesterone receptors (ER, PR) in order to explore reasons for discordant results between the two assay types. Receptor statuses in 18% of ER assays and 30% of PR assays were in disagreement. Immunohistochemistry-positive steroid-binding-negative status predominated among the discordant ER assays, while the discordant PR assays displayed the opposite situation. In discordant assays receptor concentration was significantly more often close to the cut-off (10–50 fmol mg$^{-1}$) than in the concordant ones. Low binding affinity (high $K_d$) was also significantly associated with disagreeing assay results. These observations clearly indicate that immunohistochemical ER and PR assays measure high-affinity binding components (i.e. type I receptors) in steroid-binding assays. ER but not PR assays in premenopausal women disagreed more often than those in post-menopausal women. Such factors as histological type, specimen size in steroid-binding assay, grade of malignancy and tumour necrosis were statistically unrelated to agreement or disagreement of receptor assays.

Quantitation of radiolabelled oestrogen and progestin bound to tumour extract cytosol is the established technique to assay the receptors of these hormones in breast carcinoma tissue (Jensen, 1978; McGuire & Clark, 1985). The results of such determinations have been successfully used to predict response to endocrine treatment. Moreover, steroid receptor status has been found a valuable indicator of survival and possibly of recurrence-free interval (McGuire, 1987; Fisher *et al.*, 1988; Thorpe, 1988).

Development of monoclonal antibodies to oestrogen and progesterone receptors (ER, PR) has enabled the introduction of immunohistochemical assay (King *et al.*, 1985, Perrot-Applanat *et al.*, 1987; Pertschuk *et al.*, 1988) with some potential advantages over the steroid-binding technique (King *et al.*, 1985; McCarty *et al.*, 1985; Helin *et al.*, 1988; Kinsel *et al.*, 1989). Results of the former assays, based on immunological recognition of antigenic epitopes on receptor molecules, have displayed moderate to excellent agreement with those of the radioligand binding assays (McCarty *et al.*, 1985; King *et al.*, 1985; Giri *et al.*, 1988; Helin *et al.*, 1989; Pertschuk *et al.*, 1988; Kinsel *et al.*, 1989). However, 10–30% of the results have been discordant. This study focuses on possible reasons for discordant results between steroid binding and immunohistochemical assays in 179 consecutive surgical biopsy specimens of female breast carcinoma.

### Patients, tumours and methods

### *Patients*

Surgical biopsy specimens were obtained from 179 women operated for primary breast carcinoma in 1986–1988. Median age of the patients was 61 years (range 29–92). Fifty-eight of the patients were pre- and 121 post-menopausal. All patients with amenorrhoea of more than 2 years were considered post-menopausal. When the cessation of menstruation was unknown, women at ⩾52 years of age were taken as post-menopausal.

Correspondence: H. Helin.
Received 24 October 1989; and in revised form 2 February 1990.

### *Tumours*

Breast carcinoma biopsy specimens were removed at operation and submitted for frozen section histopathological analysis. The tissue block was examined by a pathologist and divided into two portions. Both were frozen in liquid nitrogen within 15 min of removal. One portion was sectioned in a cryostat at 4–6 µm for intraoperative diagnosis, and for immunohistochemical steroid receptor analysis. For the latter, the cryostat sections were attached to microscope slides coated with poly-L-lysine (Sigma Chemical Co., St Louis, MO, USA). The other portion of the tissue block was used for radioligand binding assay of ER and PR. For both types of receptor assay, the tissues were stored air-tight at −80°C for no longer than 2 weeks.

### *Histopathological and immunohistochemical analysis*

Tumour specimens were processed to paraffin sections and stained using standard histological procedures. The tumours were histologically classified according to the World Health Organization (WHO) nomenclature (Hartmann *et al.*, 1981) and graded in three categories of malignancy as described by Blood and Richardson (1957). The grading was based on subjective estimation of tubule formation, nuclear atypism and frequency of mitotic/hyperchromatic nuclear figures. Also estimated were the occurrence of tumour necrosis, epithelium to stroma ratio within the tumour (both scored 1–3) and relative cellularity of the tumour per total section area (recorded as percentage).

Immunohistochemical determination of ER was performed with the ER-ICA immunoperoxidase kit (Abbott Laboratories, Diagnostic Division, North Chicago, IL, USA) following the manufacturer's instructions. MCF-7 cells were used as positive controls. In the negative control staining, normal rat immunoglobulin replaced the monoclonal anti-ER.

PR was detected on cryostat sections using a monoclonal mouse antibody to rabbit uterine PR (Transbio, Paris, France) known to crossreact with human PR (Logeat *et al.*, 1983). The detailed immunoperoxidase staining procedure has been described earlier (Helin *et al.*, 1988*a*, 1989). Frozen sections of rabbit uterus were used as positive controls, and negative control staining was done using non-immune mouse immunoglobulin instead of the specific antibody.

The immunoperoxidase stainings for ER and PR were semiquantitatively assessed as described by McCarty *et al.*

(1985). Each stained section was given a histoscore (hs, range 0–500) calculated from the formula: $\Sigma(i+1) \times P_i$, in which $i$ = intensity of nuclear staining (1–4, 0 = no staining) and $P_i$ = percentage of stained nuclei of carcinoma cells. On the grounds of our earlier studies, an hs $\geqslant$ 100 was considered positive for ER and an hs $\geqslant$ 40 positive for PR (Helin *et al.*, 1988*a*, 1989).

*Steroid-binding assay*

The concentrations of ER and PR in tumour extract cytosols were determined by a radioligand binding assay using multipoint titration with seven different concentrations of tritiated estradiol or Org 2058 as ligands. Dextran-coated charcoal (DCC) was used to separate protein-bound from free tritiated hormones (Vihko *et al.*, 1980). After counting of radioactivity, the binding data and dissociation constants ($K_d$) were calculated by the method of Scatchard (1949). Non-specific binding was corrected by means of a computer program (Isola *et al.*, 1988). Values equal to or higher than 10 fmol of bound $^3$H-oestrogen or progestin were regarded as positive. Assays with $K_d \geqslant 0.2$ nM (ER) or $\geqslant 0.3$ nM (PR) were scored negative except for the data in Figure 3, where the cut-off in receptor concentration was the only criterion for positivity.

*Statistical analyses*

The concordance between the immunohistochemical and steroid-binding assays was assessed with the kappa statistics (Fleiss, 1981) in which a kappa-coefficient = 0 indicates chance agreement and a kappa = 1 designates full agreement. The kappa test, one-way analysis of variance, and the $\chi^2$ tests were calculated with the Biomedical Data Processing Software Library (BMDP, Los Angeles, CA, USA).

### Results

*Comparison of immunohistochemical receptor determination with steroid-binding assay*

The two different assays, based on either radioligand binding or on immunoperoxidase location of tissue-bound monoclonal antibodies, are compared in Table I. The receptor statuses (positive or negative), defined by the two assays, agreed in 82 (ER) or 70 (PR) % of the cases. The kappa-coefficient (95% confidence limits) for ER comparisons was 0.585 (0.521, 0.649), and for those PR 0.4 (0.331, 0.469). The immunohistochemical ER assay was somewhat more often positive than the biochemical one (123 *vs* 116). Among the discordant cases, those positive in immunohistochemistry and negative in DCC assay predominated. In PR determinations, the DCC assay was a little more often positive (105 *vs* 96), and DCC-positive, immunoperoxidase-negative assay results were the predominant discordant ones (Table I).

The results of steroid-binding assays were divided into three categories (receptor concentration <10, 10–50, >50 fmol mg$^{-1}$). Discordant assay results were significantly less likely to occur with assays giving cytosol concentrations >50 fmol mg$^{-1}$ ($\chi^2$ test for linear trends: ER, $P<0.0001$; PR, $P=0.029$; Figure 1a). Similarly categorised histoscores (<100 (ER) or <40 (PR), 100–250, >250) are correlated to discordance in Figure 1b. Discordant assay results are rare in tumours with high histoscores ($\chi^2$ test for linear trends: ER, $P=0.009$; PR, $P=0.011$).

*Influence of endocrine status*

The results of the two ER assays disagreed significantly more often in premenopausal ($n=58$) than in post-menopausal ($n=121$) patients ($\chi^2$ test, $P=0.03$; Figure 2). Both types of discordance (DCC−/histo+ and DCC+/histo−) were equally represented among the premenopausal patients. In the two PR assays there was no such difference ($P=0.6$; Figure 2).

**Table I** Comparison of immunohistochemical ER and PR determinations ($ER_{hs}$, $PR_{hs}$) with cytosol steroid-binding assay ($ER_c$, $PR_c$) in 179 breast carcinomas[a,b]

| | $ER_c-$ (<10 fmol mg$^{-1}$) | $ER_c+$ ($\geqslant$10 fmol mg) | Total |
|---|---|---|---|
| $ER_{hs}-$ (<100) | 43 | 13 | 56 |
| $ER_{hs}+$ ($\geqslant$100) | 20 | 103 | 123 |
| Total | 63 | 116 | 179 |
| | $PR_c-$ (<10 fmol mg$^{-1}$) | $PR_c+$ ($\geqslant$10 fmol mg$^{-1}$) | Total |
| $PR_{hs}-$ (<40) | 52 | 31 | 83 |
| $PR_{hs}+$ ($\geqslant$40) | 22 | 74 | 96 |
| Total | 74 | 105 | 179 |

[a]Kappa-coefficient = 0.585 (ER), 0.4 (PR). [b]assays with $K_d \geqslant 0.2$ nM (ER) or $\geqslant 0.3$ nM (PR) were scored negative.

**Figure 1** Relative proportions of concordant and discordant results between steroid-binding and immunohistochemical receptor assays as functions of cytosol receptor concentration (**a**) or immunohistochemical score (**b**). Discordant results are rare in assays with high cytosol receptor concentrations (**a**) or histoscores (**b**). Note the high percentage of discordant assays in the DCC PR assay range 10–50 fmol mg$^{-1}$ (**a**). Histoscore groups: negative, <100 (ER) or <40 (PR); positive, 100–250; strong positive, >250 (**b**). Total number of tumours in each group is given above the bars. In both comparisons, the differences between the three groups are statistically significant ($\chi^2$ test for linear trends).

*Influence of dissociation constant*

The results of the radioligand assay were divided into three groups on the basis of the binding affinity (high, medium, low binding affinity), as reflected by the dissociation constant ($K_d$). Concordant results were highly significantly associated with high and medium binding affinity (low and medium $K_d$; $\chi^2$ test for linear trends: ER, $P<0.0001$; PR, $P=0.0017$, Figure 3), whereas low binding affinity was often associated with discordance between the two assays. Further, the dissociation constant was associated with the patients' endocrine status. Post-menopausal status was more likely to associate with lower $K_d$ values (higher binding affinity) than the premenopausal one ($\chi^2$ test for linear trends: ER, $P=0.0002$; PR, $P=0.0007$).

**Figure 2** Proportions of concordant and discordant assay results as functions of endocrine status. Discordant ER results are relatively more frequent in premenopausal patients ($P = 0.03$, $\chi^2$ test). In PR determinations there is no significant difference. Total number of tumours in each group is given above the bars.

**Figure 3** Proportions of concordant and discordant ER and PR assay results as functions of binding affinity ($K_d$) in steroid-binding assay ($K_d$ groups for ER: high, $<0.1$; medium 0.1–0.2; low, $>0.2$ nM; for PR: $<0.1$, 0.1–0.3, $>0.3$ nM). Concordant assays are associated with medium and high binding affinity (low $K_d$). Total number of tumours in each group is given above the bars. Cut-off in receptor concentration was the only criterior for positivity in DCC assays. The differences between the three groups are statistically significant ($\chi^2$ test for linear trends).

*Other factors*

The epithelial cell content of the tumours (reflected by the epithelium to stroma ratio) displayed a significant association with concordance in ER assays. The assay results were more likely to agree in tumours with high content of malignant epithelium (epithelium to stroma ratio score 3; $\chi^2$ test for linear trends: $P = 0.035$). Cellularity of the tumours (proportion of tumour epithelium in the microscopic section) was estimated in the frozen section (median 25%, range 5–75) and was not statistically associated with concordance.

Unrelated to the likelihood of discordance or concordance were such factors as the histologic type of the tumour, grade of malignancy and tumour necrosis. Neither was the size of the tumour specimen (median 500 mg, range 100–1,480) associated with agreement or disagreement between the assay results.

### Discussion

The results of the steroid-binding and immunohistochemical assays displayed a moderate to good agreement. The receptor status disagreed in 18 (ER) or 30 (PR) % of patients. Similar rates of concordance have been reported in ER studies by other investigators (McCarty *et al.*, 1985; King *et al.*, 1985; Hanna & Mobbs, 1989; Kinsel *et al.*, 1989). Immunohistochemical studies on PR in breast carcinoma are much fewer. Using the same monoclonal anti-PR antibody, mPRI, Perrot-Applanat *et al.* (1987) in a preliminary study, and Giri *et al.* (1988) observed a better concordance with DCC assays (92 and 86% agreement). Pertschuk *et al.* (1988) used the monoclonal anti-human PR antibody JZB 39 and found an agreement of 76 or 54% in two sets of breast carcinoma biopsy material. In our previous studies, the agreement has been 71–76% with the mPRI (Helin *et al.*, 1988*a*, 1989), and 82% with the JZB 39 antibody (Isola *et al.*, 1990).

Among the discordant assay results, the immunohistochemistry-positive DCC-negative status predominated in ER determinations. This might be due to erroneously negative results in ER DCC assays, since false positive nuclear immunostaining seems very unlikely in adequately controlled immunohistochemical analysis. Most likely to contribute to these false negative results are ER assays in premenopausal women (see below). In PR assays the predominant discordant status was the immunohistochemistry-negative DCC-positive one. One reason for these 31 discordant assay results could be insensitive receptor detection by the monoclonal antibody leading to false negative immunohistochemical results. However, we have compared the monoclonal mouse anti-rabbit PR antibody used here with the recently introduced JZB 39 anti-human PR antibody and obtained results which were only slightly in favour of the latter (Isola *et al.*, 1989).

Figure 1 in our study shows that the immunohistochemical and steroid-binding assays disagreed often when either of them yielded low receptor contents. There were virtually no discordant results when the ER concentration was $>50$ fmol mg$^{-1}$, or when ER or PR histoscores were $>250$. Eighty per cent of the results disagreed when the cytosol PR concentration was 10–50 fmol mg$^{-1}$. These results suggest that low receptor contents, especially low PR concentrations, should be taken with care as predictors of therapy response and survival. A recent prognosis study with over 1,000 patients and a follow-up of 60 months (Shek & Godolphin, 1989) demonstrated in an analysis with stratified ER DCC data that high ER levels were significantly associated with prolonged survival.

Kinsel *et al.* (1989) reported better predictive value for immunohistochemical ER determination relative to steroid-binding assay. They stated that in the steroid-binding assays more of the negative results were in the borderline range than in the immunohistochemistry. Non-receptor steroid-binding proteins or low levels of ligand binding to nonmalignant tissue components were discussed as possible reasons.

Endocrine status is clearly associated with the likelihood of discordance between the two assay types but only in ER determinations. In this study we confirmed our previous result (Helin *et al.*, 1988*b*) that ER assays are more often concordant in post-menopausal than in premenopausal patients. Interference of endogenous oestrogen with the DCC assay is one possible explanation. To support this, high plasma or serum oestradiol levels have been observed to associate with low ER concentrations (Nagai *et al.*, 1979; Helin *et al.*, 1988*b*). Other investigators have found no such association (Fishman *et al.*, 1977; Edery *et al.*, 1981). Rather than being due to occupation of ER by endogenous ligands, low ER concentrations in premenopausal patients have been examined by true down-regulation of ER synthesis in a number of studies (e.g. Saez *et al.*, 1978). The latter include demonstration of low ER mRNA levels in premenopausal breast carcinoma tissue (Barret-Lee *et al.*, 1987). Some immunohistochemical studies have also yielded lower contents of ER in receptor-positive premenopausal specimens (King *et al.*, 1985). The higher discordance between the assay results in premenopausal patients may thus partly reflect its dependence on receptor concentration.

Immunohistochemical ER and PR results correlated poorly with DCC values having high $K_d$. High $K_d$ values may be a result of erroneous interpretation of Scatchard plots or of the presence of a second steroid-binding molecule ('type II receptor'). Our results indicate that the immunohistochemical ER and PR assays recognise the high affinity steroid binding component ('type I receptor').

Potential sources of discrepancy between the two assay types and sources of error in the DCC assay are the absence of malignant tissue and receptor heterogeneity in the test sample. Thorpe (1987) and Steele *et al.* (1987) reported surprisingly high proportions of histologically unsatisfactory tumour specimens. In our study cellularity per frozen section (proportion of malignant epithelium of total sample tissue) was not related to concordance/discordance. Low epithelial cell content within the tumours was, however, significantly correlated with discordance confirming that specimens with small amounts of carcinoma cells are a problem encountered in DCC assays.

Intratumour heterogeneity in receptor expression has been demonstrated by multiple sampling in numerous DCC studies (e.g. Davis et al., 1984), and it is clearly visualised in immunohistochemical analysis. High histoscore values correspond to little heterogeneity in receptor expression and they were correlated with high degree of concordance between the two assay types in our work (Figure 1b).

Subjectivity of interpretation may interfer with semiquantitative immunohistochemical analysis similarly to conventional morphological examination. The latter has displayed unexpectedly low interobserver reproducibility in some studies on breast carcinoma histopathology (Gilchrist et al., 1985; Davis et al., 1986). Computerised image analysis may increase the objectivity of immunohistochemical analysis.

Studies focused on therapy response and survival are needed to assess the biological relevance and clinical value of immunohistochemical steroid receptor determination and its usefulness in comparison with conventional steroid-binding assay. In particular, the predictive value of receptor heterogeneity, revealed by routine immunohistochemical analysis, is of interest.

### References

BARRETT-LEE, P.J., TRAVERS, M.T., MCCLELLAND, R.A., LUGMANI, Y. & COOMBES, R.C. (1987). Characterization of estrogen receptor messenger RNA in human breast cancer. *Cancer Res.*, **47**, 6653.

BLOOM, H.J.G. & RICHARDSON, W.W. (1957). Histological grading and prognosis in breast cancer. *Br. J. Cancer*, **11**, 359.

DAVIS, B.W., GELBER, R.D., GOLDHIRSCH, A. & 8 others (1986). Prognostic significance of tumor grade in clinical trials of adjuvant therapy for breast cancer with axillary lymph node metastasis. *Cancer*, **58**, 2662.

DAVIS, B.W., ZAVA, D.T., LOCHER, G.W., GOLDHIRSCH, A. & HARTMANN, W.H. (1984). Receptor heterogeneity of human breast cancer as measured by multiple intratumoral assays of estrogen and progesterone receptors. *Eur. J. Cancer Clin. Oncol.*, **20**, 375.

EDERY, M., GOUSSARD, J., DEHENNIN, L., SCHOLLER, R., REIFFSTECK, J. & DROSDOWSKY, M.A. (1981). Endogenous oestradiol-17 beta concentration in breast tumours determined by mass fragmentography and by radioimmunoassay: relationship to receptor content. *Eur. J. Cancer*, **17**, 115.

FISHER, B., REDMOND, C., FISHER, E.R. & CAPLAN, R. (1988). Relative worth of estrogen and progesterone receptor and pathologic characteristics of differentiation as indicators of prognosis in node negative breast cancer patients: Findings from national surgical adjuvant breast and bowel project protocol B-06. *J. Clin. Oncol.*, **6**, 1076.

FISHMAN, J., NISSELBAUM, J., MENENDEZ-BOTET, C.J. & SCHWARTZ, M.K. (1977). Estrone and estradiol content in human breast tumors: relationship to estradiol receptors. *J. Steroid Biochem.*, **8**, 893.

FLEISS, J. (1969). *Statistical Methods for Rates and Proportions*. John Wiley and Sons: New York.

GILCHRIST, G.W., KALISH, L., GOULD, V.E. & 10 others (1985). Interobserver reproducibilty of histopathological features in stage II breast cancer. *Breast Cancer Res. Treat.*, **5**, 3.

GIRI, D.D., GOEPEL, J.R., ROGERS, K. & UNDERWOOD, J.C.E. (1988). Immunohistochemical demonstration of progesterone receptor in breast carcinomas. Correlation with radioligand binding assays and estrogen receptor immunohistology. *J. Clin. Pathol.*, **41**, 444.

HANNA, W. & MOBBS, B.G. (1989). Comparative evaluation of ER-ICA and enzyme immunoassay for the quantitation of estrogen receptors in breast cancer. *Am. J. Clin. Pathol.*, **91**, 182.

HARTMANN, W.H., OZZELLO, L., SOBIN, L.H. & STALSBERG, H. (1981). *Histological Typing of Breast Tumors*, 2nd edn. (eds). World Health Organization: Geneva.

HELIN, H.J., HELLE, M.J., HELIN, M.L. & ISOLA, J.J. (1988*a*). Immunocytochemical detection of estrogen and progesterone receptors in 124 human breast cancers. *Am. J. Clin. Pathol.*, **90**, 137.

HELIN, H.J., HELLE, M.J., KALLIONIEMI, O.-P. & ISOLA, J.J. (1989). Immunohistochemical detection of estrogen and progesterone receptors in human breast carcinoma. *Cancer*, **63**, 1761.

HELIN, H.J., ISOLA, J.J., HELLE, M.J. & ADLERCREUTZ, H. (1988*b*). Influence of endocrine status on biochemical and immunocytochemical estrogen and progesterone receptor assays in breast cancer patients. *Breast Cancer Res. Treat.*, **12**, 67.

ISOLA, J.J., HELLE, M.J. & HELIN, H.J. (1990). Immunocytochemical detection of progesterone receptor in breast carcinoma. Comparison of two monoclonal antibodies. *Am. J. Clin. Pathol.* (in the press).

ISOLA, J.J., OJA, S. & YLIKOMI, T.J. (1988). Computer program for correction of nonspecific binding in assays of steroid hormone receptors. *Clin. Chem.*, **34**, 431.

JENSEN, E.V. (1975). Estrogen receptors in hormone-dependent breast cancer. *Cancer Res.*, **35**, 3362.

KING, W.J., DESOMBRE, E.R., JENSEN, E.V. & GREENE, G.L. (1985). Comparison of immunocytochemical and steroid-binding assays for estrogen receptor in human breast tumors. *Cancer Res.*, **45**, 293.

KINSEL, L.B., SZABO, E., GREENE, G.L., KONRATH, J., LEIGHT, G.S. & MCCARTY, K.S. Jr (1989). Immunocytochemical analysis of estrogen receptors as a predictor of prognosis in breast cancer patients: comparison with quantitative biochemical methods. *Cancer Res.*, **49**, 1052.

LOGEAT, F., VU HAI, M., FOURNAIR, A., LEGRAIN, P., BUTTIN, G. & MILGROM, E. (1983). Monoclonal antibodies to rabbit progesterone receptor: crossreaction with other mammalian progesterone receptors. *Proc. Natl Acad. Sci. USA*, **80**, 6456.

MCCARTY, K.S. Jr, MILLER, L., COX, E.B., KONRATH, J. & MCCARTY, K.S. Sr (1985). Estrogen receptor analyses: correlation of biochemical and immunohistochemical methods using monoclonal antireceptor antibodies. *Arch. Pathol. Lab. Med.*, **109**, 716.

MCGUIRE, W.L. (1987). Prognostic factors for recurrence and survival in human breast cancer. *Breast Cancer Res. Treat.*, **10**, 5.

MCGUIRE, W.L. & CLARK, G.M. (1985). Role of progesterone receptors in breast cancer. *Semin. Oncol.*, **12**, 12.

NAGAI, R., KATAOKA, M., KOBAYASHI, S. & 5 others (1979). Estrogen and progesterone receptors in human breast cancer with concomitant assay of plasma 17beta-estradiol, progesterone and prolactin levels. *Cancer Res.*, **39**, 1835.

PERROT-APPLANAT, M., GROYER-PICARD, M.-T., LORENZO, F. & 5 others (1987). Immunocytochemical study with monoclonal antibodies to progesterone receptors in human breast tumors. *Cancer Res.*, **47**, 2652.

PERTSCHUK, L.P., FELDMAN, J.G., EISENBERG, K.B. & 8 others (1988). Immunocytochemical detection of progesterone receptor in breast cancer with monoclonal antibody: relation to biochemical assay, disease-free survival, and clinical endocrine response. *Cancer*, **62**, 342.

SAEZ, S., MARTIN, P.M., & CHOUVET, C.D. (1978). Estradiol and progesterone levels in human breast adenocarcinoma in relation to plasma estrogen and progesterone levels. *Cancer Res.*, **38**, 3468.

SCATCHARD, G. (1949). The attraction of proteins for small molecules and ions. *Ann. NY Acad. Sci.*, **51**, 660.

SHEK, L.L. & GODOLPHIN, W. (1989). Survival with breast cancer: the importance of estrogen receptor quantity. *Eur. J. Cancer Clin. Oncol.*, **25**, 243.

STEELE, R.J.C., HAWKINS, R.A., ANDERSON, T.J. & FORREST, A.P.M. (1987). The relevance of control histology in oestrogen receptor estimation. *Br. J. Cancer*, **56**, 617.

THORPE, S.M. (1987). Steroid receptors in breast cancer: sources of inter-laboratory variation in dextran-charcoal assays. *Breast Cancer Res. Treat.*, **9**, 175.

THORPE, S.M. (1988). Estrogen and progesterone receptor determinations in breast cancer. Technology, biology and clinical significance. *Acta Oncol.*, **27**, 1.

VIHKO, R., JÄNNE, O., KONTULA, K. & SYRJÄLÄ, P. (1980). Female sex steroid receptor status in primary and metastatic breast carcinomas and relationship to serum steroid and peptide hormone levels. *Int. J. Cancer*, **26**, 3.